# Impact of sleep apnea and treatments on cardiovascular disease

**DOI:** 10.5935/1984-0063.20220047

**Published:** 2022

**Authors:** Andre Faria, Arthur Macedo, Carolina Castro, Elke Valle, Raquel Lacerda, Najib Ayas, Ismail Laher

**Affiliations:** 1 Universidade Federal de Minas Gerais, Faculdade de Medicina - Belo Horizonte - MG - Brazil.; 2 University of British Columbia, Divisions of Critical Care and Respiratory Medicine - Vancouver - BC - Canada.; 3 University of British Columbia, Anesthesiology, Pharmacology and Therapeutics - Vancouver - BC - Canada.

**Keywords:** Sleep Apnea, Obstructive, Hypertension, Stroke, Atrial Fibrillation, Continuous Positive Airway Pressure, Weight Loss

## Abstract

Cardiovascular diseases (CVDs) are the leading causes of mortality worldwide, accounting for nearly 18 million deaths per year. Among other considerations, treating CVDs requires better understanding their risk factors. Sleep-disordered breathing, especially obstructive sleep apnea (OSA), is a likely contributor to several CVDs. We review key epidemiological data that addresses the link between OSA and cardiovascular outcomes such as hypertension, atrial fibrillation (AF), stroke, atherosclerosis, and heart failure (HF), and proposed pathophysiological mechanisms underlying this association. There are several biological pathways linking OSA and an increased propensity to cardiovascular diseases, and we discuss the evidence on the benefits of treatments of OSA on the prevalence of cardiovascular complications.

## INTRODUCTION

Obstructive sleep apnea (OSA) is the most common type of sleep-disordered breathing, and is characterized by repetitive partial or complete collapse of the upper airways during sleep^1^. This phenomenon reduces or prevents airflow and causes frequent arousals, and has several metabolic and physiological consequences^2,3^. The diagnosis of OSA is ideally made using overnight polysomnography (PSG) with 12 recording channels and specialized professional observation and data analysis, although there are lower cost alternatives, such as portable monitoring devices, that are also validated diagnostic methods^4^. The severity of OSA is determined by the number of apneas and hypopneas per hour of sleep and is quantitated using the apnea-hypopnea index (AHI) (see Table 1).

**Table 1:** Classification of the severity of OSA.

Apnea/hypopnea index (AHI)	
**AHI score**	**Classification**
<5 events/hour	Normal
5-15 events/hour	Mild
16-30 events/hour	Moderate
>30 events/hour	Severe

Cardiovascular diseases (CVDs) are the leading causes of mortality worldwide, accounting for nearly 18 million deaths per year. Common CVDs include functional abnormalities in the cardiac and vascular system that can result in heart failure, strokes, arrhythmias, atherosclerosis, hypertension, and cardiac ischemia^5^. Although the comorbidities and risk factors for these conditions are numerous and diverse, OSA frequently occurs in patients with CVDs, in part, due to the global epidemic of obesity^6-8^. The metabolic syndrome (MS) is one of the most important risk factors for the development of OSA (OR=2.87/95%CI=2.41 3.42)^8^. The MS is an umbrella term describing a constellation of conditions, such as obesity, hypertension, insulin resistance, and hyperlipidemia that not only increases the risk of OSA but also of CVDs^9^.

Even though OSA and cardiovascular outcomes often present in partnership, their association can reflect either a cause/effect relationship or as different endpoints having similar risk factors. The coexistence of these conditions does not prove causality, and potential confounding variables should be considered. In addition, treating OSA can also effect the prevalence of CVDs. This review summarizes our current understanding of the relationship between sleep apnea and CVDs based on current evidence.

### Pathophysiological mechanisms linking OSA and cardiovascular diseases

**Figure 1:**
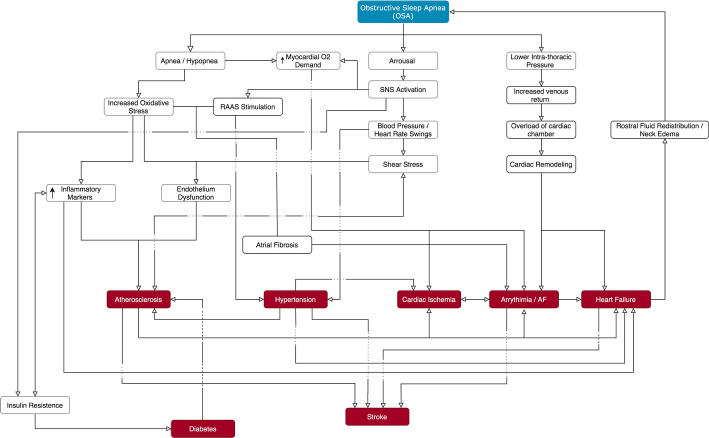
Pathophysiological mechanisms underlying the development of CVDs in OSA.

Abbreviations: CVDs = Cardiovascular diseases; SNS = Sympathetic nervous system; RAAS = Renin-angiotensin-aldosterone system.

OSA is associated with a multitude of cardiovascular pathological mechanisms (see Figure 1). Sleep apnea is related to oscillations in intrathoracic pressure caused by repeated inspiratory efforts against a collapsed upper airway^10^. Intrathoracic pressure becomes very negative during apneas, and increases right heart volume due to a greater venous return^11^. The substantial changes in ventricular transmural pressure increases cardiac wall stress (afterload) and atrial enlargement, leading to cardiac remodeling and arrhythmias^12-16^.

OSA is also associated with increases in oxidative stress, which contributes to atrial remodeling and arrhythmic pathologies. Sleep apnea promotes the production of systemic inflammatory proteins and the activation of the sympathetic nervous system by cyclical arousals, leading to swings in blood pressure (BP) and heart rate in patients with OSA^17-20^. This phenomenon generates shear stress and, combined with hypoxia, leads to dysfunction of the vascular endothelium^10^. The combination of hypoxia and enhanced sympathetic nerve activity increases myocardial oxygen demand, cardiac ischemia and atrial fibrillation^21,22^.

The augmented sympathetic nerve discharge increases BP in individuals with OSA during sleep, which is in contrast to nocturnal decreases that usually occur in those without OSA^23,24^. However, activation of the sympathetic nervous system and increases in blood pressure in patients with OSA are not restricted to the sleep state, but can also occur during wakefulness, and can trigger left ventricular systolic dysfunction^25-27^. These OSA induced stressors generate long-t erm electrical and mechanical remodeling of both atria and ventricles, and eventually can lead to heart failure^28-31^, although the onset of heart failure can also increase vulnerability to OSA^31^. Patients with heart failure accumulate fluid in the lower limbs during the day while in an upright position; this increases venous return when they lay down to sleep, resulting in rostral fluid redistribution and neck edema that exacerbates OSA^10^.

Atherosclerosis is an important cause of myocardial infarction that can also lead to heart failure. OSA can contribute to atherosclerosis via multiple mechanisms such as increased oxidative stress, systemic inflammation, vascular smooth cell/lymphocyte activation, augmented adhesion molecule expression, enhanced lipid accumulation by macrophages, and endothelial dysfunction^32^. Consequently, OSA also predisposes cerebrovascular accidents, as many of the risk factors for stroke (such as hypertension, autonomic dysfunction, oxidative stress, endothelial dysfunction, atherosclerosis, arrhythmias, and hypercoagulability) are increased in OSA patients^33^.

An added factor is that intermittent hypoxia increases insulin resistance due to decreases in the phosphorylation of tyrosine kinases and the effectiveness and sensitivity of insulin receptors^34,35^. The overactivation of the sympathetic system leads to reduced secretion of insulin and hyperglycemia as well, especially due to a reduction of pancreatic P-cell metabolism^34,35^.

Importantly, the irregular and incomplete sleep patterns of OSA patients causes drowsiness during the day, weight gain, deposition of adipose tissue and ectopic fat, besides decreased activity and energy expenditure. These alterations exacerbate obesity, so aggravating dyslipidemia, insulin resistance and hyperglycemia, increasing the risk of developing cardiovascular diseases^35,36^.

### Hypertension

The association between OSA and systemic arterial hypertension is a subject of intense investigation. OSA and hypertension often coexist, with the prevalence of OSA in hypertensive patients varying between 30-50%^37,38^. In patients with drug-resistant hypertension this number increases to 70%, with OSA being by far the most common secondary associated condition^37,38^. A longitudinal study that analyzed data from the Wisconsin sleep cohort study concluded that sleep-disordered breathing increased the risk of developing hypertension by ~3fold, even when confounding factors such as age, gender, body habitus, smoking, alcohol use, education, and physical activity were accounted for. Having higher AHI values strengthens the association between OSA and hypertension^39^. A recent systematic review and meta-analysis reported a significant association of essential hypertension with mild (OR=1.18, 95%CI=1.09-1.27, *p*<0.05), moderate (OR=1.32, 95%CI=1.20-1.43, *p*<0.05) and severe OSA (OR=1.56, 95%CI=1.29-1.84, *p*<0.05)^40^. For studies comparing OSA and non-OSA participants, the pooled OR was 1.80 (95%CI=1.54-2.06, *p*<0.05)^39^. Finally, there is a pooled OR of 2.84 (95%CI=1.70-3.98, *p*<0.05) in six studies that explored the association between OSA and resistant hypertension^40^.

It is important to note that no significant association was reported between hypertension and OSA in patients aged 60 years, although the reasons for this are unclear. It could be related to a selection bias and the cross-sectional nature of the studies^1,41^. Two alternative hypotheses could be the different phenotypic expression of sleep apnea related to the proportion of mixed and central apneas, which can impact the underlying pathophysiology, and the co-existence of other medical conditions affecting the interaction between OSA and systemic hypertension in the elderly^41,42^.

### Atrial fibrillation

Atrial fibrillation (AF) occurs frequently in patients with OSA^15,18,43,44^, as both conditions share several risk factors such hypertension, congestive heart failure, and coronary artery disease, hampering the determination causal relationships^44^. Multivariate analysis reports a strong independent association between the odds of having OSA and developing AF (OR=2.2)^45^. A multicenter cohort study reports that the chances of developing cardiac arrhythmias are three times greater in patients with OSA compared to patients with no sleep-disordered breathing (OR=4.02, 95%IC=1.03-15.74)^46^. The strength of the proposed causal link is strengthened by a meta-analysis indicating that AF is prevalent in OSA patients (OR=2.12, 95%CI=1.85-2.44, *p*<0.001)^47^.

Higher AHI scores increase the susceptibility to AF, with an odds ratio of 2.91 in men with an established OSA diagnosis^48^. Reversal of AF reversal is associated with an increased risk of recurrence of AF after one year (82% vs. 42%, *p*=0.01) in those untreated for OSA^49^.

The higher prevalence of OSA in patients with AF also suggests that sleep apnea could initiate and perpetuate arrhythmias^15,18,43^. A recent prospective cohort study of patients with AF reported an OSA prevalence of 85%, which is significantly higher than the prevalence of 18.2 % reported in other studies^50,51^. One reason for these differences in prevalence of AF may be that OSA may be under-recognized in some arrhythmic patient groups, as patients with cardiac arrhythmias are not routinely screened for OSA^50,51^.

### Cerebrovascular disease

Recent studies propose OSA as an independent risk factor for cerebrovascular diseases, and it is estimated that 3070% of people who have suffered a stroke also have OSA^52-55^. A meta-analysis of 12 prospective cohort studies (25,760 subjects) suggests an increased risk (2.15 greater) of incident fatal and non-fatal strokes in patients with severe OSA^56^. Another meta- -analysis also supports this assertion, even after adjustment for confounding factors such as age, sex, BMI, diabetes, hypertension, coronary artery disease, previous coronary artery intervention, heart failure, smoking, alcohol use and dyslipidemia (RR=1.94; 95%CI=1.31-2.89; *p*=0.001)^55^.

The relationship between OSA and stroke was also highlighted by a meta-analysis of 10 prospective studies showing that OSA was associated with a ~2.1-fold higher risk for fatal or non-fatal stroke, increasing to a ~6.4-fold higher risk in untreated patients (AHI>10 events/hour) over a median of 6.8 years of follow-up^57^. Taken together, these results indicate an enhanced risk of stroke in patients with OSA that occurs independently of other risk factors and which is related to OSA severity. The absence of effective OSA treatments can have a detrimental effect on stroke risk^57^.

### Atherosclerosis

Coronary computed tomography angiography images indicate that the severity of OSA increases both non-calcified/ mixed coronary plaques and total arterial stenosis scores. Patients with severe OSA have 3.8- times (95%CI=1.46-10.40; *p*=0.01) greater risk of non-calcified/mixed plaques independent of other risk factors, so increasing their vulnerability to acute coronary syndrome^58^.

Early markers of atherosclerosis in hypertensive and OSA groups suggest that carotid intima-media thickness and carotid diameter is increased in individuals with either OSA or hypertension, but that these were exaggerated in patients with both OSA and hypertension, where there was also decreased carotid distensibility^59^. These results suggest additive effects OSA and hypertension on the progression of atherosclerosis and that OSA is independently associated with carotid intimamedia thickness^59^.

### Heart failure

Patients with heart failure (HF) frequently report sleepdisordered breathing, and pathophysiological links between these conditions lead to decompensation of heart failure in OSA patients^60^. However, some suggest that the high prevalence of OSA in patients with HF may be related to demographic criteria (age, sex, ethnicity, and race), similar risk factors (obesity and comorbid conditions), AHI cutoff, and severity of HF^61,62^.

A cross-sectional analysis of 6,424 adults from the Sleep Heart Health Study indicates that OSA is associated with a 2.38 increase in the relative odds of presenting with HF^61^. This association is reinforced by two different studies of patients with OSA confirmed by polysomnography^60,63^. These studies report that 21% of individuals with HF and preserved ejection fraction (HFpEF) have OSA, 11% of individuals with HF and reduced ejection fraction (HFrEF) have OSA, and 7.7% of all OSA patients have left ventricular ejection fraction <50%^60,63^. Despite the positive findings proposing a strong association between OSA and HF, large RCTs with longer follow-up periods have not been reported.

### Influence of OSA treatment on cardiovascular consequences

The current gold-standard treatment for OSA is the continuous positive airway pressure (CPAP) device, which consists in a mask that is attached to a pump that forces air into the upper airways through the nasal passages. The continuous pressure during both inspiration and expiration must be sufficient to overcome the structural obstruction in the upper airways. The majority of studies of OSA treatment is based on interventions with CPAP (see Table 2), although there are also other approaches such as lifestyle changes (e.g., reduction of BMI, avoidance of alcohol and sedatives before bedtime), surgical procedures (specially uvulopalatopharyngoplasty - UPPP), oral appliances, and other devices for enlargement of nostril orifices (see Table 3).

**Table 2:** Evidence of CPAP effects in cardiovascular diseases.

Cardiovascular disease	Observations made based on CPAP therapeutical effects
Hypertension	Minimum CPAP use of at least 4 hours is able to reduce BP pressure in all profiles of hypertension^64^ CPAP also reduces BP in resistant hypertension patients, with a better response proportional to OSA severity^65-72^.
Atrial fibrillation	CPAP reduces AF development risk in 42% and its recurrence (42% vs. 82%)^73-76^. CPAP also reduces the risk of progression to permanent AF (HR=0.66)^76,77^.
Cerebrovascular disease	A recent RCT indicates that patients who were adherent to CPAP therapy had a lower risk for stroke (HR=0.56) and for composite endpoint of cerebral events (HR=0.52)^78^. These results were not adjusted for multiple testing, and post hoc CPAP dose-response analysis showed no significant associations^78^. A different RCT suggests no benefit of CPAP treatment on cerebrovascular risk reduction^79^.
Atherosclerosis	A RCT showed that CPAP treatment reverses markers of atherosclerosis, decreases carotid intima-media thickness, pulse-wave velocity, C-reactive protein, and catecholamines after 4 months^80^. It is also shown a reduction in tumor necrosis factor-a (TNF-a) and interleukin-6 (IL-6)^81^. Withdrawal of CPAP treatment exacerbates endothelial dysfunction in patients with minimally symptomatic OSA^82,83^.
Heart failure	CPAP usage shows some positive outcomes, such as improvements in LVEF and pulmonary pressure in 12 weeks. Among patients with LVEF lower than 45%, CPAP led to an improvement in LVEF from 25 to 35% (*p*<0.001) and reduction in systolic blood pressure by 10mmHg (*p*=0.03) after a month of treatment^84-86^. Nonetheless, this treatment reduces biochemical markers of HF (sympathetic activity)^87^. There are still some questions about the effects of CPAP on the survival rate. Some studies show improvement, as the risk for death and hospitalization was increased in the untreated group (HR=2.03) and in less compliant CPAPtreated patients (HR=4.02) when compared to the compliant CPAP-treated group^88^. Meanwhile, other studies don’t show improvements in this matter^84,89^. Still, HF patients with OSA treated with CPAP continue to experience rostral fluid shifts during sleep, increasing neck circumference^31^.

Abbreviations: CPAP = Continuous positive airway pressure; BP = Blood pressure; OSA = Obstructive sleep apnea; AF = Atrial fibrillation; HR = Hazard ratio; RCT = Randomized controlled trial; LVEF = Left ventricular ejection fraction; HF = Heart failure.

**Table 3:** Evidence of alternative OSA treatments.

Treatment	Description	Observations made on its therapeutical effects
Lifestyle changes	A combination of behavioral interventions, aiming weight loss (through dietary changes and exercising), sleep hygiene and avoidance of alcohol and tobacco consumption.	Several meta-analysis and systematic reviews indicate that lifestyle changes can improve OSA primary outcomes, such as AHI, oxygen desaturation, and excessive daytime drowsiness^94-99^. A recent RCT shows reductions of BP, independently and associated with CPAP therapy, being stronger in the combined approach^100^. Additionally, growing evidence supports weight loss can reduce AF burden and arrhythmia related complications^18,101,102^. Weight loss and frequent exercises are also related to reducing platelet reactivity and aggregation, reducing atherosclerosis and stroke risk^103^.
Oral appliance therapy (OAT)	Oral appliances to treat OSA fall into two broad categories: tongue retaining devices and mandibular advancement splints (MAS). MAS are extensively used and the predominant category. The appliance aims to slightly advance the mandible forward and enlarge the upper airway. It also prevents the collapse of the throat passage. There are several designs and the selection of the most appropriate model, besides its degree of advancement and fitting, require a special training and a qualified professional.	Hypertension: two different studies reported that oral appliances led to slight reductions in mean 24-hour and awake BP, measured with 24-hour ambulatory blood pressure monitoring (ABPM), restricted to hypertensive patients^104,105^. Andrén et al. (2013)^108^ replicated the same results, however the ameliorations were observed only in moderate-severe OSA patients, after 3 months of treatment^107^. Contrariwise, Trzepizur et al. (2009)^109^ did not find any changes in blood pressure (measured with a finger monitor) after 2 months with either oral appliance or CPAP therapy in treated hypertensive patients^110^. Atrial fibrillation: to date, there is a lack of good quality data assessing the effect of OAT on cardiac arrhythmias. Cerebrovascular disease: Anandam et al. (2013) reported a reduction in cardiovascular mortality, including stroke, after treatment with OAT^110^. However, the fatal events were a composite endpoint (stroke, myocardial infarction, sudden cardiac arrest and arrhythmias) and independent results for stroke were not available. Atherosclerosis: A recent RCT found no significant changes in most oxidative stress parameters after 1 month of oral appliances for moderate OSA patients^109^. Parallelly, a different study found that inflammatory markers (high sensitivity C-reactive protein, and fibrinogen) were reduced after 3 months, and 1 year of treatment with oral devices in mild to moderate OSA patients^110^. It is also shown that oral appliances resulted in a significant improvement in endothelial function after a treatment period of 2 months, and significant reductions in arterial stiffness after 1 month of oral appliance and CPAP therapy^105,107^. Heart failure: Two studies assessed left ventricular mass and did not find any effect of oral appliance on this heart function parameter after 3 months of treatment. No further results were found^111,112^.
UPPP	The most common OSA surgical procedure is uvulopalatopharyngoplasty (UPPP). The surgery consists in removing excess of tissue from the back of the throat (tonsils, uvula, and part of the soft palate).	The benefits of UPPP on reducing OSA parameters and improving CVDs are extremely limited and no consistent data was found for the majority of conditions. For hypertension, a systematic review was found describing reductions in blood pressure in 5 studies (two as a primary outcome and 3 as a second)^113^.

Abbreviations: OSA = Obstructive sleep apnea; AHI = Apnea/hypopnea index; RCT = Randomized controlled trial; BP = Blood pressure; CPAP = Continuous positive airway pressure; AF = Atrial fibrillation; CVDs = Cardiovascular diseases.

### Continuous positive airway pressure (CPAP)

There is much evidence that CPAP reduces blood pressure and AF progression/recurrence in patients with OSA, but the benefits of CPAP in stroke (risk and/or progression) is unclear^74,79^. There are many advantages of CPAP treatment in preventing atherosclerosis, although RCT studies and metaanalysis are needed to investigate changes in blood pressure and inflammatory markers by CPAP.

Most studies on the impact of CPAP on LVEF and other cardiac markers in HF are 1-3m onths in duration^87,90,91^. The reported amelioration of HF in RCTs should thus be evaluated with caution. The improvements noticed with CPAP therapy were assessed by measuring fatigue, drowsiness and humor, with no analysis of exercise capacity or shortness of breath (dyspnea). Improvements in the latter markers would better characterize improvements in cardiac insufficiency resulting from HF in OSA.

Despite some positive results, additional studies are still needed to access the benefits of CPAP on preventing CVD development and progression. Results may vary in their findings of CPAP benefits, likely due to differences in sample sizes, study designs, confounding variables, gender, OSA diagnostic methods, and variability in the duration and compliance of the CPAP treatments^70^.

The sleep apnea cardiovascular endpoints (SAVE) study, an international, multicenter, randomized trial evaluated the long-term benefits of CPAP on cardiovascular events in 2,717 OSA patients. CPAP and control groups were matched for age, gender and BMI. The SAVE study reported that CPAP treatment failed to improve cardiovascular outcomes and did not prevent progression to more chronic conditions such as heart failure^78^. These results suggest that CPAP therapy should not be used as a primary treatment for protection against CV events or reducing mortality^78^, and also supported by a more recent systematic review of the poor efficacy of CPAP therapy in reducing cardiovascular risk^92^. Nevertheless, it is important to note that there are other benefits of CPAP therapy, such as improvements in sleep quality and daytime function, in patients with cardiovascular diseases^93^. In addition, it must be recognized that the patients in the SAVE study were in general not sleepy and CPAP adherence was low; these may have also contributed to the null results^94,95^.

### Alternative strategies

The literature describing the effects of lifestyle changes on cardiovascular outcomes and OSA is scarce and inconclusive. Despite overall favorable improvements in measures of sleep apnea, possible benefits of CVD in OSA are unclear except for reductions in blood pressure in such patients. Studies of the comparative, additive or independent benefits of weight loss and lifestyle modifications in obese OSA patients with cardiovascular comorbidities are unavailable. It is likely that improvements were likely related to the direct effects of lifestyle modifications on CVD outcomes rather than on reducing the impact of OSA on cardiovascular pathologies. Nevertheless, the clinical benefits of reductions of blood pressure and OSA parameters (AHI, daytime sleepiness, and O2 desaturation) due to lifestyle modifications have not been reported in RCTs with long follow-up periods.

Studies on the effects of uvulopalatopharyngoplasty (UPPP) on blood pressure report mixed results^113^. Potential reasons for this may be that some studies failed to adjust for the use of antihypertensive medications, had differences in outcomes such as UPPP success rates and frequency of blood pressure measurement (office-based blood pressure measurements were predominantly used, rather than home or ambulatory measurements). Importantly, the majority of studies were case reports or cohort studies with limited sample sizes.

Several recent publications report some benefits of surgical procedures on AF^18,115^. Renal sympathetic denervation (RSD) shows promising effects on reducing AF^18,115^, possibly due to reductions of arrhythmogenic atrial autonomic, structural and electrical remodeling; RSD is as efficient as combined therapy with beta-blockers and angiotensin receptor blockers^116^. These findings are mostly from isolated pre-clinical animal studies; clinical studies in OSA populations are needed to establish RSD as an alternative approach.

The positive outcomes of oral appliance therapy (OAT) in the treatment of OSA are mostly inconclusive. Comparative studies on cardiovascular outcomes using different therapies for OSA are relatively scarce^116^, although a cohort study reports that oral appliances are as effective as CPAP in reducing cardiovascular death (HR=1.08)^108^, although this is inconsistent with other findings that show that CPAP does not alter cardiovascular mortality^78,92^.

Although small reductions in blood pressure are important (every 5mmHg reduction in the mean systolic BP reduces cardiovascular risk by 17%), the minor reductions observed in many studies using alternate therapies for OSA (ranging from-0.07 to-0.77mmHg) may have limited clinical impact^105-107,109,117-119^, as they were based on the findings using relatively short follow-up periods (1-3 months) with small patient sample sizes^105-107,109^.

Two studies reported improvements in arterial stiffness and endothelium function with OAT^106,107^. Only few studies evaluated the effects of OAT circulating cardiovascular biomarkers of OSA^110,111,119^; these studies were generally underpowered and evaluated a variety of outcomes, such as oxidative stress parameters (thiobarbituric acid reactive substances, erythrocyte superoxide dismutase activity, uric acid, homocysteine, folate, and vitamins B12 and E dosages) and inflammatory markers (high sensitivity C-reactive protein, and fibrinogen). More extensive studies are needed to elucidate the effects of OAT on circulating cardiovascular biomarkers of OSA and their clinical relevance to atherosclerosis and cerebrovascular disease. The evidence describing the therapeutic potential of OAT on CVD outcomes in OSA is still at its infancy. Additional studies directly comparing cardiovascular event rates are important, but will probably require RCTs with large patient groups and also long-term follow-up studies.

## CONCLUSION

Our understanding of OSA and its association with CVDs continues to evolve. This brief survey of the literature indicates that OSA both predisposes to and aggravates hypertension, atrial fibrillation, and cerebrovascular disease. The relationship between OSA and atherosclerosis and HF, while being plausible, needs more extensive investigation. It is difficult to establish a causal effect of OSA on CVDs, as these are related in a multidirectional manner. Future studies should focus on risk stratification of OSA patients related to adverse cardiovascular outcomes. Studies to identify new OSA-specific biomarkers of cardiovascular risk are needed.

Additionally, there is a need for large-scale, randomized, well-controlled studies on the effects of CPAP on cardiovascular diseases such as hypertension, atrial fibrillation, stroke, atherosclerosis, and heart failure that are unhindered by issues of poor compliance.

There are only a few studies on the modest benefits of alternative therapies for OSA on reducing CVDs in OSA. The clinical benefits of surgical treatment of OSA on CVD in OSA are unclear. Lifestyle changes can improve sleep-apnea parameters and hypertension. However, the degree of their influence (additive or independent) and whether these changes are clinically relevant is unclear. Oral appliances likely reduce blood pressure, atherosclerosis and stroke in OSA, but more rigorous large scale RCTS are needed. Long-term populational studies that directly compare cardiovascular event rates with different management protocols of OSA would be informative.
